# What is left in miombo woodlands? Rarity and commonness of woody species, commercial timber species, and lawful harvestable diameter classes

**DOI:** 10.1016/j.heliyon.2025.e41821

**Published:** 2025-01-09

**Authors:** Tarquinio Mateus Magalhães

**Affiliations:** Departamento de Engenharia Florestal, Universidade Eduardo Mondlane, Av. Julius Nyerere Número 3453, Campus Universitário Principal, Edifício Número 1, 257, Maputo, Mozambique

**Keywords:** *Brachystegia*, Charcoal, Deforestation, Firewood, Forest logging, *Julbernardia*, Species rarity

## Abstract

Mozambican miombo woodlands (MWs) have been experiencing severe anthropogenic threats, recognized to have an impact on plant species distribution, occurrence, diversity, and rarity patterns. Based on 3725 0.1 ha plots distributed across the country's MWs, this study aimed to assess the species rarity and commonness, protection status, and availability of commercial timber in MWs under varied environmental conditions. Results show that, out of the 515 tree and shrub species found, 45 % were rare, while just 10 % were highly protected. Nine of the 112 commercial miombo species were not observed, and 15 were rare. Commercial-sized trees of the top nine desired species were extremely rare, some species had only 1 tree per 20 ha and stem forms unsuitable for sawmilling. Selective overharvesting has also affected trees with no minimum felling diameter. *Brachystegia spiciformis*, *B. boehmii*, and *Julbernardia globiflora* stood out among the few timber species with commercially viable populations. MWs in the semi-arid ecoregion have lower species richness, fewer commercially viable populations, and a higher number of poorly protected species (51 %). The rainy ecoregion has the highest percentage of poorly protected species (61 %). Based on the results obtained, it was recommended that (1) timber harvesting should be restricted to the humid ecoregion, prohibited for the timber species that were either absent or rare, and halted for the top nine desired species; (2) except *B. spiciformis, B. boehmii*, and *J. globiflora*, MWs should preferably undergo a forest closure period corresponding to at least one cutting cycle; (3) to ensure long-term viability, a minimum harvestable density for commercial-sized trees should be determined for each species.

## Highlights

• Almost 50 % of Mozambican miombo woody species are rare.
• Only 10 % of the miombo woody species are highly protected.
• Commercial-sized trees of desirable species are scarce.
• The remaining commercial-sized trees of desirable species have poor forms for milling.
• *Brachystegia* spp. and *Julbernardia* spp. are the few with commercially viable stocks.

## 1 Introduction

The miombo region spans 11 nations in eastern and southern Africa and covers an area of 2.7–3.6 million km^2^ [1]. Around 100 million people live in the miombo region and rely on it for income and consumption [[1], [2], [3]]. Miombo woodlands (MWs) are important globally for carbon sequestration and as centres of endemism [4]. The miombo-mopane woodlands are one of the five worldwide ecozones that need to be prioritized for biodiversity protection because they are unreplaceable in terms of species endemism [5]. It has been estimated that out of the 8500 plant species found in the miombo region [2], 4590 are unique to it [1], and of the more than 300 tree species, 54 % are endemic [2].

MWs contain relatively few good commercial timber species, the best regions have long been logged over, the remaining commercially viable stocks are limited and difficult to access, and most MWs are extensively degraded ecologically [2]. The average annual rate of woodland loss in the miombo region varies between 0.2 and 1.7 % [1]. Fuelwood and charcoal production, clearing for shifting cultivation, and forest logging are the main drivers of miombo degradation, deforestation and species loss [1,2,[6], [7], [8]].

MWs cover approximately 60 % of the forested area of Mozambique [9] and are home to five centres of endemism [10]. With the arrival of Chinese timber operators in Mozambique in the early 2000s, forest logging increased considerably, and by 2010, timber had become Mozambique's sixth largest export product, accounting for 3 % of total exports [11]. In the same period, urbanization and population expansion raised wood fuel use by 14 % for every 1 % rise in urbanization [12]. The need for land for agriculture has grown along with the population. There are roughly 21 million rural residents in Mozambique [13], with 19 million^1^ of them thought to reside in the country's miombo region and depend on it for subsistence. The latter is expected to reach 35 million^1^ by 2050, putting additional pressure on the woodlands.

Between 2003 and 2013, the average annual deforestation rate was 0.79 %, ranging from 0.16 in Maputo province to 4.35 % in Nampula province [14]. Forest conversion to shifting cultivation fields and forest conversion to grassland as a result of forest logging and biomass extraction for energy accounted for 86 and 13 % of deforestation, respectively [14]. Between 2007 and 2009, the volume of charcoal production increased from 273548 to 470725 m^3^ [15], six times more than the annual allowable cut (AAC) reported by Marzoli [16]. Wood harvesting became heavily concentrated on just three timber species (*Afzelia quanzensis, Millettia stuhlmannii*, and *Pterocarpus angolensis*) [17,18], accounting for 58 % of the total harvest and 85 % of the total wood consumption in the country [15,17,18]. These timber species have been documented to be exploited above their ACC [18]. One of the most serious and common anomalies was the harvesting of trees that had not achieved the minimum harvestable diameter [17,18]. From 2007 to 2013, on average, 81 % of forestry exploitation in Mozambique was illegal and in 2013 alone, 93 % of timber logged was illegal and 76 % of timber exported came from illegal logging [19].

The aforementioned anthropogenic threats are recognized to have an impact on the distribution, occurrence, diversity, and rarity patterns of plant species [[20], [21], [22], [23], [24], [25], [26]]. Therefore, the general goal of this study was to establish a framework for defining priority miombo woody species for conservation based on Rabinowitz's [27] forms of rarity and protection status, as well as to assess the availability of commercial-sized timber species and trees. To achieve this goal, the following research questions were set: based on Rabinowitz's [27] framework, how rare or common are the miombo species? What is the current status of miombo species protection? How numerous are the merchantable timber species? What is the frequency of occurrence of commercial-sized trees? Should all merchantable timber species, regardless of the ecoregion and stem density, continue to be logged? Which measures and restrictions should be enforced to ensure sustainability and prevent the collapse of miombo ecosystems?

## 2 Material and methods

### 2.1 Study area

The study area comprises the MWs of Mozambique (10° 00′ to 26° 52′S and 30° 12′ to 40° 51′E). Mozambique is a country located on the south-eastern coast of Africa, bordering South Africa, Eswatini, Zimbabwe, Zambia, Malawi, and Tanzania, with an Indian Ocean coastline of 2700 km and an area of 799380 km ^2^, of which 786380 km ^2^ is land area and 13000 Km ^2^ corresponds to the water area [28]. Forests and woodlands cover 41 % (326068 km^2^) of the land area, with MWs accounting for approximately 60 % [9].

The country's average rainfall per year, over the last 40 years, ranges from 800 to 1000 mm along the coast, with values above 1200 mm between Beira and Quelimane [28]. The rainfall decreases inland reaching 400 mm at the border with South Africa and Zimbabwe [29]. The north and central part of the country has an annual rainfall from 1000 to over 2000 mm because of the northeast monsoon and high mountains [29]. In the southern inland part of the country, the average annual rainfall ranges from 500 to 600 mm [29].

### 2.2 Sampling design and field measurements

**Fig. 1:**
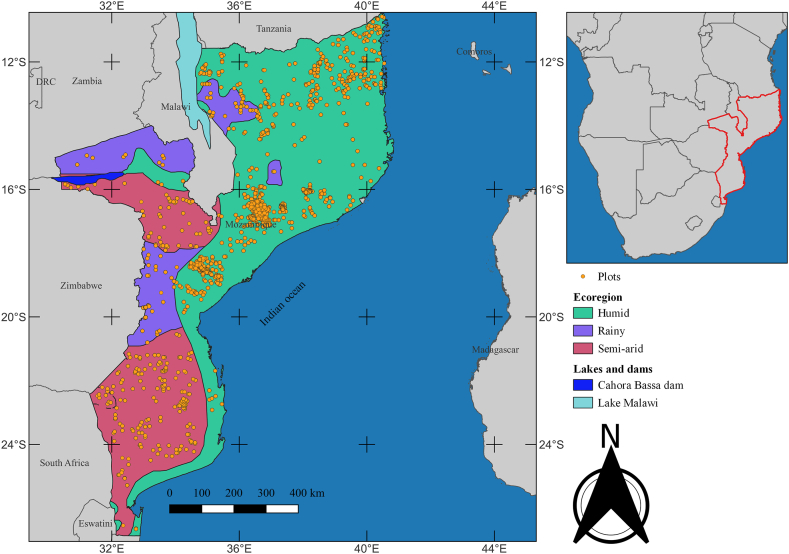
Distribution of sampling units into miombo habitats.

MWs were divided by habitat to allow for species classification according to habitat specificity. Climate was used to categorize the habitats as humid, semi-arid, or rainy. Humid and semi-arid habitats have humid tropical and semi-arid climates, respectively, whereas rainy habitats have an altitude-modified climate. According to CHIRPS (Climate Hazards group InfraRed Precipitation with Stations) data, the mean annual rainfall in semi-arid, humid, and rainy ecoregions, respectively, has ranged from 300 to 700, 800 to 1200, and over 1200 mm over the last 41 years. These numbers align with those from other sources and historical periods [[28], [29], [30]]. MWs were, therefore, divided into those found in humid, semi-arid, and rainy environments (Fig. 1).

**Fig. 2:**
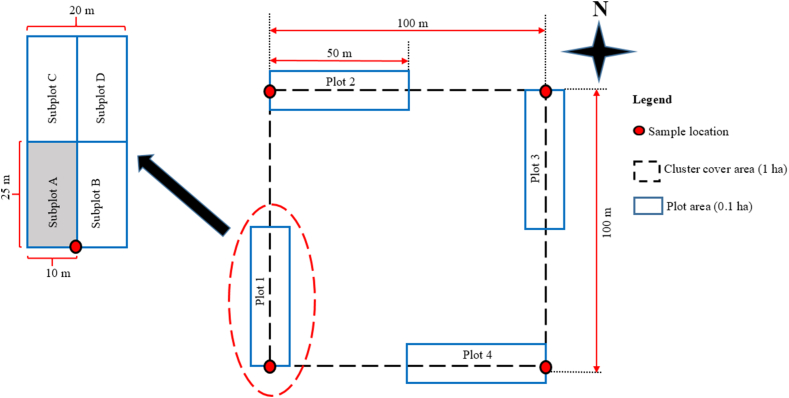
Cluster plot design. Cluster cover area is the area of the geographic figure formed by the lines (dashed lines) between the sample locations of the plots, the extent of the ground area enclosed by the boundaries of a cluster.

The data for this study were gathered from the most recent national forest inventory [31] as well as from several regional and restricted area forest inventories that employed the national forest inventory methodology [[32], [33], [34], [35], [36]]. The sampling units were 1 ha square clusters with four 50 × 20 m plots established in the clusters' corners (Fig. 2). The plots were further divided into four 25 × 10 m subplots (Fig. 2). Within the plots, all trees and shrubs having a diameter at breast height (DBH) greater than or equal to 10 cm were measured for DBH and were scientifically recognized. All shrub and tree species in each plot were identified in the field at the species or subspecies level by the botanists from the National Botanical Garden and Eduardo Mondlane University Botanical Garden. Plant specimens were gathered and sent to the herbaria of the botanical gardens described earlier to identify species that could not be identified in the field. Non-woody species that were occasionally mistaken for woody species (in the field or the herbarium) were identified and excluded from analyses based on their scientific names.

Because there are often more small trees than large ones in natural forests, fixe-area sampling results in a sample with many small trees and fewer large trees [37,38]. This makes tree measurement arduous and time-consuming. To address this issue and make sampling less labour-intensive and time-consuming, trees with DBH ranging from 5 to 10 cm were only examined within the bottom left subplot (Fig. 2, subplot A). All trees with DBH larger than 10 cm were assessed based on stem form and scored from 1 to 3 as follows. SF1 (stem form 1): straight trunk with no significant deviation from the central axis, perfect for sawlog. SF2: waving stem, trunk slightly deviated from the central axis, with small bends. SF2 form is acceptable but not ideal for milling purposes, can be partially used for sawmilling. SF3: crooked stem, with severe bends, unsuitable for sawmilling.

The sample size was 3725 plots (Fig. 1), which were proportionally distributed as follows—2696, 722, and 307 in humid, semi-arid, and rainy ecoregions, respectively. There were 880 plots within the protected areas (PAs) and 2845 outside. Here, 76, 80, and 73 % of plots assigned to humid, semi-arid, and rainy ecoregions, respectively, were outside PAs. The remaining 24, 20, and 27 % were in PAs.

### 2.3 Computation and analyses

#### 2.3.1 Diversity, species richness and composition

The number of tree and shrub species per habitat, or species richness index, was utilized to evaluate species diversity. Additionally, two nonparametric species richness estimators, bias-corrected Chao 1 and ACE (abundance-based coverage estimator) were used to detect changes in species richness between habitats [39,40].

The Sørensen dissimilarity index [41] was used to estimate β-diversity and hence quantify the disparity in terms of species composition between pairs of habitats (pair-wise β-diversity, pair-site Sørensen dissimilarity). Multiple-site Sørensen dissimilarity index was used to evaluate the overall β-diversity (all habitats together). Additionally, β-diversity was partitioned into two components— turnover (measured as Simpson dissimilarity) and nestedness (measured as a nestedness-resultant fraction of Sørensen dissimilarity) [42,43]. This made it possible to ascertain whether the difference in species composition between ecoregions was mostly due to species replacement or species loss.

The “Betapart” R package (version 1.6) [44] was used to compute and partition β-diversity into turnover and nestedness components. “Vegan” package (version 2.6–4) [45] was used to extrapolate species richness based on Chao 1 and ACE indices.

#### 2.3.2 Commonness or rarity of woody species

The commonness or rarity form of each woody species was determined using the Rabinowitz framework [27,46]. Rabinowitz classified species rarity based on geographical distribution, habitat specificity, and local population size (abundance) (Table 1). Additionally, the Rabinowitz framework-based indices developed by Maciel [47] (geographical range index, habitat specificity index, population size index, and rarity index) were utilized to calculate the geographic and numerical rarity as well as habitat specificity of each species.

**Table 1:** Species classification into eight categories using Rabinowitz's [27] framework.

Geographic distribution	Habitat specificity (preference)	Population size (abundance)	Rarity form
Wide (>10 %)	Various (>1)	Large (abundance >2)	Common
		Small (abundance ≤2)	Form 1
	Single (1)	Large (abundance >2)	Form 2
		Small (abundance ≤2)	Form 3
Narrow (≤10 %)	Various (>1)	Large (abundance >2)	Form 4
		Small (abundance ≤2)	Form 5
	Single (1)	Large (abundance >2)	Form 6
		Small (abundance ≤2)	Form 7

The "Rare7″ R package [48] was used to categorize each woody species based on Rabinowitz's seven forms of rarity, and the "rrindex" R package [47] was used to compute Maciel's rarity indices.

#### 2.3.3 Protection status

Woody species and plants found within protected areas (PAs) are more likely to be well protected than those found outside. The protection status of woody species can, thus, be assessed by comparing species occurrence within and outside of PAs [[49], [50], [51]]. Therefore, the protection index (PI) suggested by Maciel et al. [50], which is the ratio of the number of occurrences within PAs to the total number of occurrences, was used to assess the status of protection. This was done at the habitat level and for the overall MWs. PI values closer to 0 indicate that the species is poorly protected and hence more vulnerable to anthropogenic activities, whereas PI values closer to 1 indicate that the species is highly protected and thus less vulnerable to anthropogenic activities [50]. In this study, however, three levels of protection status were considered in accordance with Maciel and Martins [49]—species occurring exclusively within PAs (PI = 1) and species occurring solely outside PAs (PI = 0) were defined as highly and poorly protected, respectively. Whereas, species found in both within and outside PAs (0 < PI < 1) were referred to as partially protected. Furthermore, the Sørensen dissimilarity index was also used to evaluate the dissimilarity in species composition between MWs within and outside the PAs.

#### 2.3.4 Commercial availability of timber species

Forest logging is restricted by three factors—species (whether it is categorized as a merchantable timber species), location (whether it is inside or outside of a PAs), and legal harvestable diameter. To determine how scarce and abundant commercial-sized timber species and trees are, their proportion of occurrence and relative abundance was evaluated at the habitat level, focusing on these trees outside of PAs.

## 3 Results

### 3.1 Diversity, species richness and composition

**Fig. 3:**
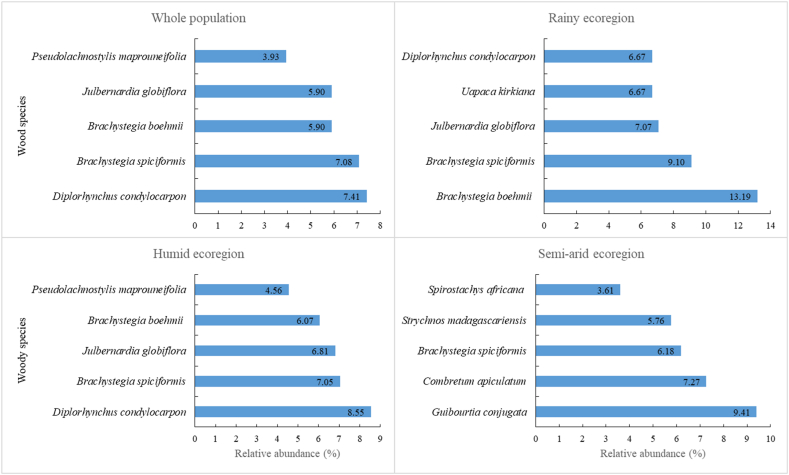
Top five woody species in terms of abundance by habitat and across the entire the miombo woodland.

A total of 103980 trees and shrubs were recorded, representing 515 species from 217 genera and 64 families. Fabaceae was the most numerous family, with 131 species, hence accounting for 25 % of all species. Overall, the number of occurrence records for each species ranged from 1 to 7703 (Supplementary material, Table S1). In the entire MWs, 35 species have only been observed twice, 52 have only been observed once, and 21 have been observed more than 1000 times (Supplementary material, Table S1), accounting for approximately 60 % of all trees and shrubs observed. Ninety percent (90 %) of the 515 woody species identified had a stem density of less than 1 ha^−1^. The top 5 most commonly observed woody species were *Diplorhynchus condylocarpon* (7703 records), *Brachystegia spiciformis* (7357), *Brachystegia boehmii* (6139), *Julbernardia globiflora* (6135), and *Pseudolachnostylis maprouneifolia* (4089). Fig. 3 provides the top five most abundant species per ecoregion. Of the total number of trees and shrubs recorded in MWs across the country (103980), 79, 17 and 9 % were found in humid, semi-arid, and rainy ecoregions, respectively, comprising 450, 271, and 233 woody species, respectively. The Chao1 and ACE indices also estimated a greater number of species in humid habitats (Chao1 = 474 ± 10, ACE = 475 ± 10) than in semi-arid (Chao1 = 292 ± 9, ACE = 301 ± 8), and rainy habitats (Chao1 = 260 ± 12, ACE = 254 ± 8).

**Fig. 4:**
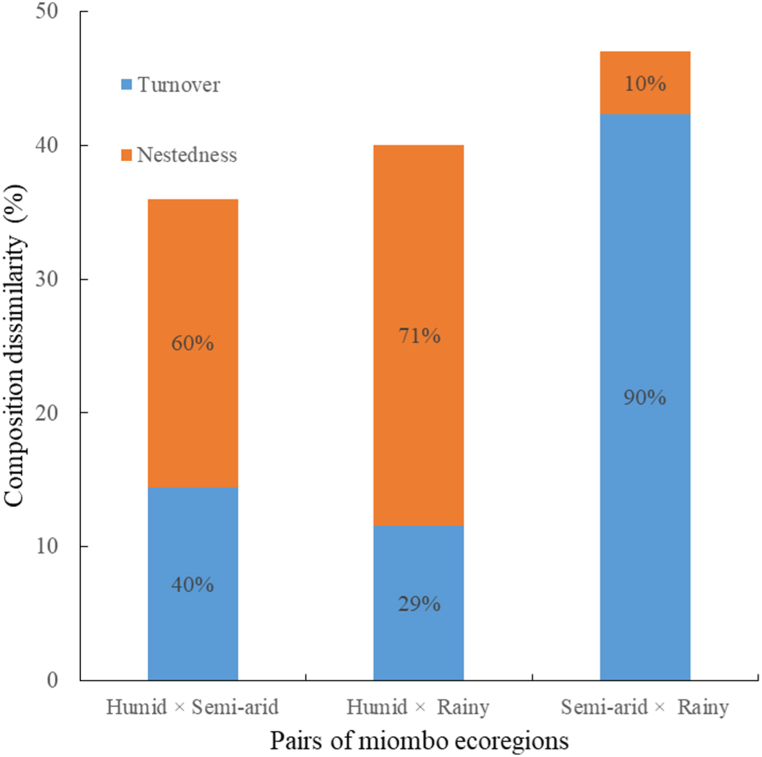
Total dissimilarity in species composition (β-diversity) and its components (turnover and nestedness) between pairs of three miombo ecoregions.

MWs in semi-arid and humid ecoregions had the largest composition dissimilarity (Fig. 4), with species turnover accounting for 90 % of the variation in composition. This indicates that species replacement, rather than species loss, was the primary driver of species composition variations between these two miombo communities. The nestedness component accounted for the majority of the dissimilarity between humid and rainy and between humid and semi-arid ecoregions (Fig. 4). This suggests that the main cause of the differences in species composition between these two pairs of miombo ecoregions was species loss. Multiple-site β-diversity revealed a species composition dissimilarity of 46 % across all ecoregions.

### 3.2 Species rarity and commonness

**Fig. 5:**
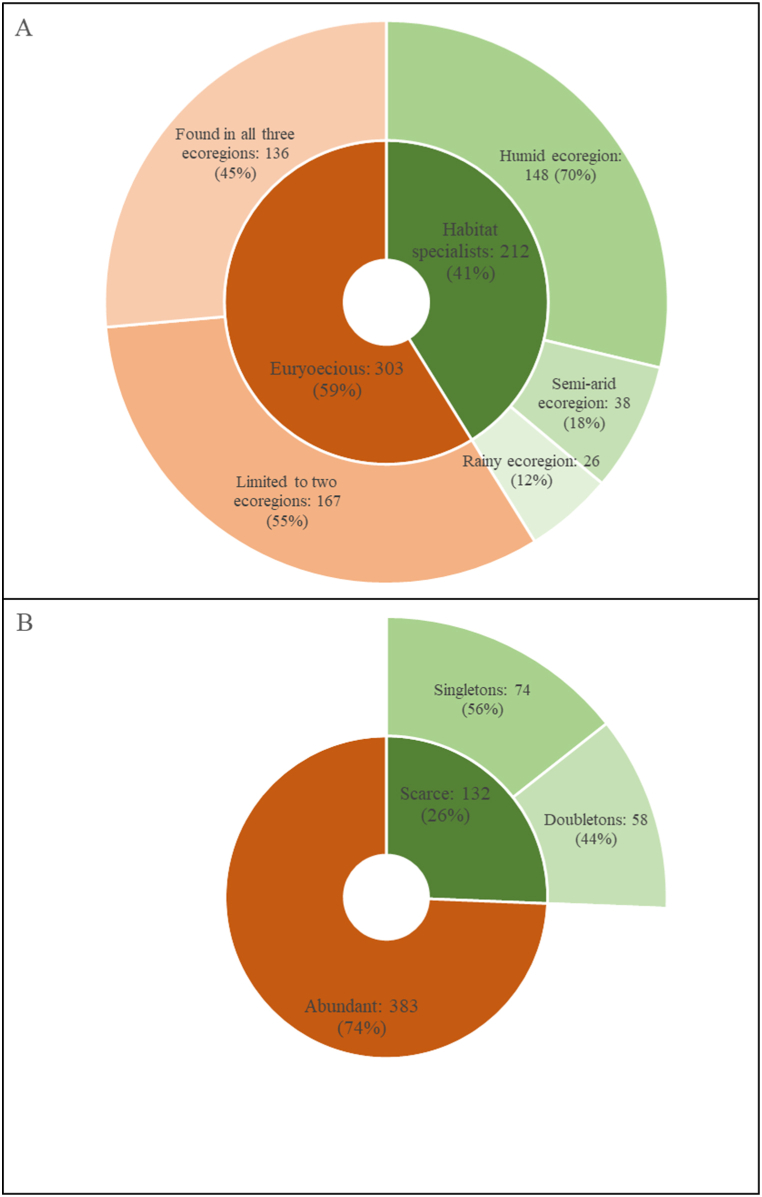
Miombo species quantification by (A) habitat specificity and (B) population size.

**Fig. 6:**
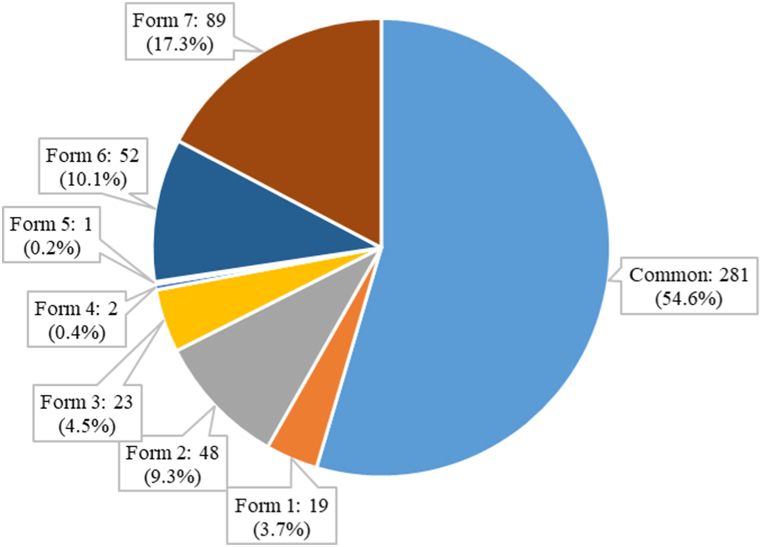
Miombo species quantification based on based on the Rabinowitz's [27] framework.

In terms of habitat specificity (Fig. 5A, Supplementary material, Table S2), the humid ecoregion is home to the majority of habitat specialists (stenoecious species). The majority of euryoecious species (those found in more than one habitat) are restricted to two ecoregions, with only 45 % being fully generalists. In terms of population size (Fig. 5B), around three-quarters of the scarce species are singletons (species represented by only one individual in their habitat). Two-thirds of the scarce species appeared only once or twice across the MWs, regardless of habitat. Taking into account the geographic range, it was found that 33 % of the total woody species are stenotopic (geographically restricted) and the remaining are eurytopic (widely distributed in the study area, geographically common). When the three rarity criteria (geographical range, local population size, and habitat specificity) were combined using the "Rare7″ R package [43], it was shown that 55 % of all species are common and 45 % are rare. The rare species are categorized into seven forms of rarity, as presented in Fig. 6. Of the 234 rare species, 15 are merchantable timber species. Rarity form 7 represents the extreme form of rarity (species that are simultaneously stenotopic, stenoecious and scarce).

### 3.3 Protection status

**Fig. 7:**
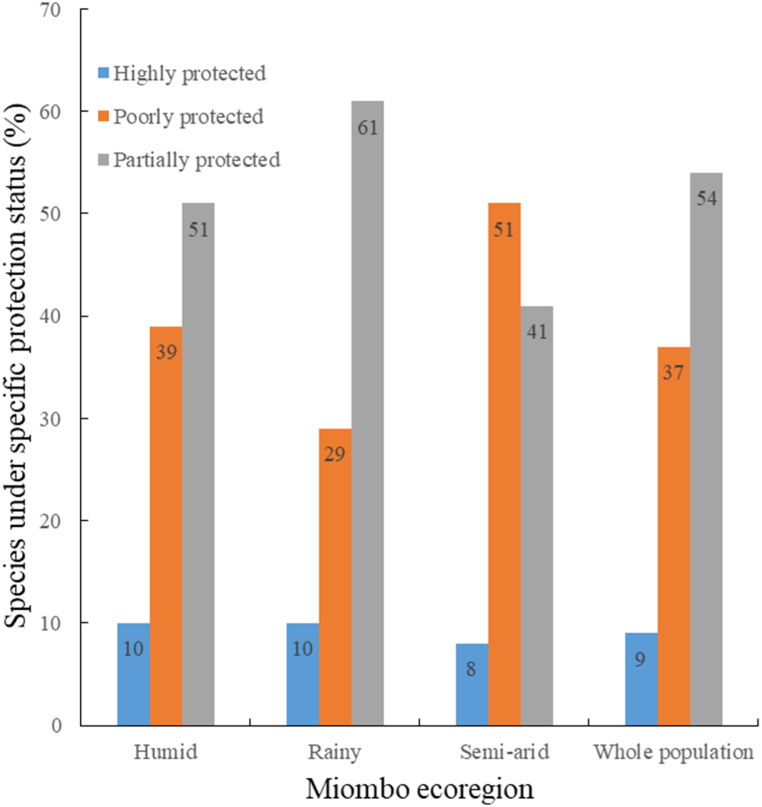
Miombo species quantification by protection status.

PI indicated that highly protected species are rare in MWs (Fig. 7). Rainy and semi-arid ecoregions are dominated by poorly protected species (Fig. 7). In humid ecoregion, however, most of the species are partially protected (Fig. 7). As a result, composition dissimilarity between MWs within and outside PAs was higher in rainy (55 %) and semi-arid (42 %) environments and lower in humid environments (30 %). For the entire MWs, the compositional dissimilarity between MWs within and outside PAs was 30 %.

The majority of the 234 rare species (66 %), as well as the majority of the 212 habitat specialist species (66 %), were found to be poorly protected. Similarly, 69 % of the 132 singleton and doubleton species discovered were poorly protected. The majority of the 281 common species (86 %), including the key miombo species, were found to be partially protected, as they were located both inside and outside PAs. Of the 44 highly protected species, 43 fall into rarity forms 6 and 7, with only *Xylia mendoncae* being a common species.

As per the IUCN Red List of Threatened Species [52], this study identified one critically endangered species (*Fernandoa magnifica*), five nearly threatened species (*Lannea schweinfurthii, Erythrophleum lasianthum, Pseudobersama mossambicensis, Milicia excelsa*, and *Dalbergia melanoxylon*), and four vulnerable species (*Khaya anthotheca, Monodora stenopetala, Xylia mendoncae*, and *Cynometra carvalhoi*). *Craibia brevicaudata* is classed as nearly threatened, vulnerable, and endangered, respectively, for its three subspecies—*brevicaudata, schliebenii*, and *burttii*. This species could not be classified under the IUCN threat categories since the study could not identify it down to the subspecies level.

According to Rabinowitz's [27] framework, nine of the abovementioned species are common and two are rare, falling into rarity forms 6 (*Craibia brevicaudata*) and 7 (*Fernandoa magnifica*). *Xylia mendoncae* and *Craibia brevicaudata* were found to be highly and poorly protected, respectively, with the remaining species classified as partially protected.

### 3.4 Commercial timber species

Excluding species from other forest types (mangrove and mecrusse), Mozambican MWs are made up of 112 legally classified timber species (*Colophospermum mopane* included) [53,54]. Of those 112 timber species, nine were not observed, 15 were classified as rare (Supplementary material, Table S2), and 87 (79 %) were found to be common. The timber species with no records include *Morus lactea, Podocarpus falcatus, Cassipourea gummiflua, Celtis gomphophylla, Funtumia latifolia, Kigelia pinnata, Vachellia erioloba* (former *Acacia erioloba*), *Borassus aethiopiocum*, and *Treculia africana*. *Parkia filicoidea, Guibourtia coleosperma*, and *Sapium ellipticum* occurred only once or twice in the entire MWs.

Up to 20 % of the number of trees and shrubs recorded were found within PAs. Outside the PAs, 50–68 % of the trees and shrubs recorded were timber species. Commercial-sized timber species trees accounted for 2, 3, and 5 % of all trees and shrubs found in semi-arid, rainy, and humid environments, respectively, which corresponded to stem densities of 6, 10, and 16 ha ^−1.^

Here, 91 of the 112 commercial miombo species were identified in humid environments, of which 63 had trees with harvestable diameters. Just four of these species had a stem density ≥1 ha ^−1^: *B. spiciformis* (4 ha ^−1^), *B. boehmii* (2 ha ^−1^), *J. globiflora* (1 ha ^−1^), and *P. maprouneifolia* (1 ha ^−1^). Moreover, the stem densities of 46 out of the 63 species (73 %) were less than one tree per 5 ha. In semi-arid environments, 72 commercial timber species were discovered, 41 of which had harvestable trees. None of the 41 timber species had a stem density ≥1 ha ^−1^, including species of genera *Brachystegia* and *Julbernardia*. Thirty-two (32) of the 60 timber species found in rainy ecoregion had harvestable trees. Only three of the 32 timber species had a stem density ≥1 ha ^−1^: *B. spiciformis* (3 ha ^−1^), *B. boehmii* (2 ha ^−1^), and *Uapaca kirkiana* (1 ha ^−1^).

In Mozambique, only a few preferred miombo woody species are targeted for logging—*Afzelia quanzensis, Berchemia zeyheri, Dalbergia melanoxylon, Combretum imberbe, Guibourtia conjugata, Millettia stuhlmannii, Pterocarpus angolensis, Spirostachys africana,* and *Swartzia madagascariensis*. These species' harvestable diameter trees accounted for 0.19, 0.60, and 0.65 % of all trees found in semi-arid, rainy, and humid ecoregions, respectively, which corresponded to stem densities of 1.6, 0.7, and 2.3 ha ^– 1^. These trees were only found in 307 (8 %) of the 3725 plots that were observed. None of these species exhibited a stem density of more than two individuals per 5 ha in any of the ecoregions, except *P. angolensis* and *S. africana*, which had four and three trees per 5 ha of humid and semi-arid environments, respectively. For *P. angolensis* in semi-arid habitats, *C. imberbe* in humid habitats, *G. conjugata*, *S. madagascariensis*, and *C. imberbe* in rainy habitats, there were no commercial-sized trees. Furthermore, only 47 % of these species' stems had a stem form suitable for sawmilling (SF1 trees). The majority of the trees with DBH <20 cm (71 %), on the other hand, had an adequate stem form for sawmilling.

The top three most preferred timber species out of the nine described above are *A. quanzensis, M. stuhlmannii*, and *P. angolensis* [11,17,55]. Despite providing 85 % of the wood used in the country, these species are becoming harder to find in MWs [17,18]. The harvestable diameter trees of these species together accounted for 0.03, 0.13, and 0.43 % of all trees and shrubs observed in semi-arid, rainy, and humid environments, respectively. In semi-arid, rainy, and humid habitats, the stem densities of *A. quanzensis* trees with harvestable diameter were 1, 2, and 5 per 20 ha, respectively, whereas that of *M. stuhlmannii* was 1, 2, and 6 per 20 ha, respectively. For the MWs of rainy and humid environments, the stem density of commercial-sized *P. angolensis* trees was 5 and 16 per 20 ha, respectively. Here, 72, 63, and 51 % of *M. stuhlmannii, A. quanzensis*, and *P. angolensis* harvestable trees had stems unsuitable for sawmilling (SF3 trees). The minimum felling diameter for these species is 40 cm. When only trees in the DBH class of 10–20 cm were considered, it was discovered that less than 28 % of the total trees had a stem form inappropriate for sawmilling (SF3 trees), with the majority (67 %) being SF1 trees (trees with stem form suitable for sawmilling). However, in the DBH class of 20–30 cm, SF3 trees predominated (52 %).

Of the 1003 legal forest loggers in 2018 [56], only two harvested species other than those mentioned above, primarily *B. spiciformis* and *J. globiflora*. In this study, *B. boehmii*, *B. spiciformis*, and *J. globiflora* were among the top five most abundant woody species. Despite this, the commercial-sized trees of these species accounted for just 0.33, 1.45, and 2.12 % of all trees and shrubs observed in semi-arid, rainy, and humid ecoregions, respectively, equivalent to stem densities of 0.8, 5.0, and 7.0 ha^–1^. The majority of the trees (73 %) of these species had stems suitable for sawmilling (SF1 form). The minimum felling diameter for these species is 40 cm. The SF1 trees accounted for 74, 72, and 69 % of the trees in the diameter classes of 10–20, 20–30, and 30–40 cm, respectively. Conversely, SF2 trees comprised 17, 19, and 7 % of the trees in the aforementioned diameter classes, while SF3 trees accounted for the remaining 9, 9, and 24 %.

## 4 Discussion

### 4.1 Species rarity, commonness, and protection status

The majority of the species in rainy and semi-arid ecoregions were found to be poorly protected, and the species composition dissimilarity between MWs within and outside PAs was greatest in these regions. Consequently, the species composition of MWs inside PAs is not representative of that of MWs outside PAs. This can be explained by the small number of PAs with MWs in rainy and semi-arid regions. There are just three PAs in Mozambique's rainy regions—Chimanimani National Park (CNP), Gorongosa National Park (GNP), and hunting concession 13. The latter are partly situated in rainy areas. Only Mount Gorongosa, which is part of GNP, is located in a rainy zone. The difference in species composition between woodlands within and outside PA is furthered by the fact that CNP and Mount Gorongosa are dominated by mountain evergreen forests rather than MWs. Although there are three national parks (Zinave, Banhine, and Limpopo) and seven hunting concessions in semi-arid regions, MWs are primarily found in Zinave National Park [57]. Banhine and Limpopo national parks feature mecrusse and mopane woodland types [58,59]. While some species are shared by mopane and miombo woodlands [60], a higher degree of species composition similarity is not guaranteed by the shared percentage of species.

Miombo woodlands cover all or part of the 31 PAs in humid regions, which include four parks, four national reserves, nine forest reserves, 11 hunting concessions, and three community PAs. The reduced dissimilarity is a result of the several PAs that increase the likelihood of species composition similarity between MWs inside and outside of the PAs. Lower dissimilarity (higher similarity) occurs from shared species between MWs within and outside of PAs, resulting in a higher number of partially protected species.

The majority of the rare species were discovered to be poorly protected (found only outside PAs). Rare species are inherently more vulnerable to extinction [47,48]. This susceptibility is significantly increased if the species is poorly protected (found only outside PAs). This is because the exposure to anthropogenic activities, such as shifting cultivation, is higher outside PAs. Shifting cultivation is the primary cause of deforestation in MWs [14], and it occurs often and on a significant scale outside of PAs.

The species *Xylia mendoncae* is endemic to Mozambique and is listed as vulnerable on the IUCN Red List of Threatened Species [61]. However, based on Rabinowitz's [27] framework, it was categorized as common in this study, because it was found in two habitats (humid and semi-arid) and had an abundance greater than two. It was also designated as a highly protected species because it was found solely within PAs: hunting concession 4 in Manica province, hunting concessions 11 and 12 in Sofala province, and the buffer zone of GNP in Sofala province. However, Matimele et al. [61] indicated that this species is restricted to Inhambane province, namely the districts of Inhassoro, Govuro, and Vilankulo.

*Fernandoa magnifica* is a critically endangered species that is said to be endemic to Tanzania's Rondo Forest Reserve [62], however, it was found in this study. It was found to be rare, habitat-specific (humid ecoregions), and partially protected. In reality, this species is legally categorized as commercial in Mozambique [53,54]. It was discovered in the current investigation in Sofala province, both outside and inside PAs (hunting concessions 11 and 12).

Approximately half of the woody species identified were rare. Although it is typical of natural forests for the majority of trees to belong to a small number of common species, with local rare species accounting for the majority of species richness [63,64], these rare species deserve special attention because they are more vulnerable to extinction [47,48]. This extinction risk is heightened for anthropogenically threatened woodlands (e.g., MWs). Shifting cultivation poses a serious threat to MWs [14,65]. Research indicates that under tree-based shifting cultivation, the trees remaining in agricultural fields are the key miombo species (*Brachystegia* spp. and *Julbernardia* spp.) [[66], [67]], which are common [61,62]. This means that rare species are more likely to be eradicated from tree-based shifting cultivation fields. Therefore, rare species serve as a basis for defining priority species for conservation [48], especially for Mozambican MWs where the majority of the rare species (66 %) were found to be poorly protected.

### 4.2 Timber species

Semi-arid environments are primarily found in Gaza and Inhambane provinces, which have the smallest rural population [13], alternative woodlands (mecrusse and mopane) other than MWs [[68], [69], [70]], and the least pronounced poverty, compared to those provinces of central and northern Mozambique [71,72]. Despite this, MWs of semi-arid environments are the poorest in terms of commercially viable stocks, merchantable timber species, and species richness and are also the most impacted by human activity. This is because semi-arid woodlands are fragile [73], and semi-arid ecoregions suffer from water scarcity, threatening agricultural production [[74], [75], [76]]. This situation raises the pressure on local woodlands for biomass harvest for energy. The firewood and charcoal consumed in Mozambique's southernmost towns originate from semi-arid woodlands, and 70 % of households in the semi-arid zones produce charcoal [77,78].

Agriculture is the main source of revenue for rural people in humid ecoregions, whereas biomass harvesting for energy is the main source of income in semi-arid environments such as Gaza and Inhambane. Agriculture replaces MWs in humid habitats, and the remnant woodlands suffer less from biomass harvesting than those in semi-arid regions. In semi-arid regions, however, biomass harvesting for energy induces woodland depletion rather than land cover change. Charcoal and fuelwood production results mostly in the removal of trees, whereas agriculture results in the removal of woodlands. This explains why remnant MWs of humid environments outperform those of semi-arid environments. Furthermore, while most forest concessions are in humid environments [79], licenses for charcoal and firewood exploitation are largely in semi-arid areas. Forest concession holders do not permit the manufacturing of firewood or charcoal on their properties.

It was discovered that the desirable timber species with harvestable trees exhibited generally an unsatisfactory stem form for sawmilling, but the majority of trees in the smaller diameter class (10–20 cm) demonstrated an ideal stem form for sawmilling. This shows that MWs have been depleted of commercial-sized trees of desirable timber species and that the only commercial-sized trees that remain are those that were rejected during forest logging because they were not suited for sawmilling. The DBH class just below the minimum-felling DBH (20–30 cm) had also been selectively overharvested for *M. stuhlmannii, A. quanzensis*, and *P. angolensis* species, as most of the trees had an inadequate stem morphology for milling.

Except *B. spiciformis, B. boehmii, J. globiflora*, and *P. maprouneifolia* found in humid ecoregions, and *B. spiciformis, B. boehmii* found in rainy ecoregions, commercial-sized timber trees are rare and usually with inappropriate stem for sawmilling. As a result, it is strongly advised that MWs undergo a forest closure period corresponding to at least one cutting cycle for all merchantable timber species, especially for the most desired, except the species indicated above in the appropriate ecoregions. The term "forest closure" refers to the cessation of forest logging in this context. MWs of semi-arid environments are fragile [73,80], lack commercially viable stocks, have a higher proportion of poorly protected species (51 %), and are under pressure for charcoal and firewood production [77,78]. On the other hand, compared to other ecoregions, MWs of rainy environments, have the largest proportion of poorly protected species (61 %) and occur mostly in high elevations with steep hillslopes [9]. Therefore, forest logging of any timber species should be limited to MWs of the humid ecoregion.

The cutting cycle for Mozambican MWs is defined to be 40 years. However, studies conducted in Ghana and the Amazon have shown that forests do not recover their initial volume stock in a 40-year cutting cycle [81,82]. Other studies have suggested a 100-year cutting cycle to fully recover the initial volume stock [[83], [84], [85]]. Therefore, even 40 years might not be sufficient for MW recovery. Thus, after 40 years, a new study must be carried out to decide whether to continue or interrupt the forest closure.

It is suggested that commercial timber species be reclassified to give greater value to species of the genera *Brachystegia* and *Julbernardia*. *B. spiciformis, B. boehmii*, and *J. globiflora* are currently classed as being in the second commercial class. It is also suggested that commercial timber species that were not observed in the 3725 plots and those found to be rare be removed from the list of commercial timber species.

The proposal to promote secondary timber species, such as those of the genera *Brachystegia* and *Julbernardia*, to alleviate the few species in high demand on the market that have become scarce, dates back to the early 2000s [55], with subsequent insistence [17,18], but without concrete actions. It is claimed that the lack of data to support the ideas stopped them from being implemented. Figures and evidence are compiled here to support such ideas and any quick action.

Despite having trees with harvestable diameters, a commercial timber species may not be commercially available because of low stem density or rarity. Rare species are more vulnerable to both natural and anthropogenic disturbances, such as overexploitation, habitat loss, and global environmental changes [22,[86], [87], [88]]. Therefore, it is proposed that in addition to the minimum felling diameter, a minimum harvestable stem density for commercial-sized trees be legally established for each timber species. Furthermore, a species cannot be harvested if it exhibits any sort of rarity (geographical rarity, numerical rarity, or habitat specificity). A timber species will be harvested only if its harvestable trees meet a minimum legal stem density and if it is a habitat generalist, and numerically and geographically common.

The scarcity of some tree species, particularly commercial ones, is not solely attributable to the rampant exploitation of MWs over the last >20 years. It is, in fact, a combined action of MW nature and overexploitation. MWs support relatively few commercial timber species [2]. Overall, most trees in natural forests belong to a small number of common species, with locally rare species accounting for the majority of species richness [63,64]. In this study, it was shown that only 21 of the 515 woody species identified accounted for 60 % of all trees and shrubs observed and that 90 % of the 515 woody species detected had a stem density less than 1 ha^−1^.

### 4.3 Study limitations and future scope

Cluster plots were utilized as sampling units in this study, with stratified random sampling as the sampling design, with miombo ecoregions serving as the strata. The cluster was made up of four 0.1 ha rectangular plots placed in the corners of a 100 m × 100 m area (cluster cover area), which meant that the sample locations of the plots were 100 m apart (Fig. 2). Based on the study's goal of assessing the rarity, commonness, abundance, and protection status of miombo woody species, the question that arises is: how effective was the sampling and plot design in capturing rare species?

Surveys of rare species are challenging due to the difficulty of detecting them on a landscape [89]. One of the most used methods for estimating species richness, composition and abundance is the fixed-area sampling [37,38], which can be divided into single plot design, cluster plot design, and strip design [37,38,89,90].

Several studies [[90], [91], [92]] have shown that cluster plot designs are more efficient, accurate and precise in estimating species richness, diversity, composition, and abundance than single plot designs. The spatial heterogeneity of forests, such as miombo woodlands, has a significant impact on the efficiency and performance of cluster plots. This is because, by spreading plots apart, a cluster plot could potentially capture a large variety of local plant species [91], increasing the accuracy and precision of the estimated species richness [91,93].

It has been discovered that the more apart the plots of a cluster are, resulting in a bigger cluster cover area, the more successful the cluster is at estimating species richness and composition and capturing unique species [[90], [91], [92]], despite the fact it increases travel costs between the plots [91]. This is due to the fact that when a cluster's cover area grows, the spatial autocorrelation between its plots reduces [94,95]. The cluster cover area of this study is ten times bigger (10000 m^2^) than that recommended by Zhao et al. [90] and Quon et al. [91] for tropical forests, implying better precision and accuracy in estimating species richness and composition and in capturing rare species.

It is important to note, nonetheless, that, everything else being equal, more precise and accurate estimates of species richness and composition could have been attained by expanding the cluster cover perimeter while preserving its area (e.g., from 100 m × 100 m to 200 m × 50 m). This is because a wider perimeter leads to lower autocorrelation between plots [96].

Overall, fixed-area sampling is commonly recognized as prone to missing rare species [97]. Floristic habitat sampling [98] and adaptive cluster sampling [99] are reported to be more efficient in capturing rare species [89] than fixed-area sampling. However, floristic habitat sampling is known to be less accurate in determining abundance than a fixed-area sample [98]. Moreover, it does not appear to be appropriate for national forest inventories because it calls for skilled investigators to carefully survey every possible meso and microhabitat by walking through the study area until the searcher is satisfied that every habitat has been sufficiently surveyed [89,98].

Adaptive cluster sampling is notable for effectively sampling rare species and providing reliable abundance estimates [99,100]. However, it is limited to clustered species [99,101], which is not the case for the rare species found in this investigation. Acharya et al. [101] found that, compared to fixed-area sampling, adaptive cluster sampling was up to 500 % more efficient in estimating the abundance of clustered species but 40 % less efficient for unclustered species.

Additionally, both floristic habitat sampling and adaptive cluster sampling target identified rare species, and the fieldwork is focused on searching for them. In this study, however, the objective was to identify which species are rare in miombo woodlands, without any prior knowledge of their rarity. Therefore, cluster plot design appears to be the most appropriate under the given study region and circumstances.

## 5 Conclusions

Approximately half of the miombo tree and shrub species are rare, with two-thirds being poorly protected. Commercial-sized trees of the desired timber species were extremely rare and with stem forms unsuitable for sawmilling. Trees and shrubs with no minimum-felling diameter have also been impacted by selective overharvesting. Miombo woodlands of semi-arid environments have lower species richness and are the poorest in terms of commercially viable stocks than those of humid and rainy environments. *B*. *spiciformis, B*. *boehmii*, and *J*. *globiflora* stood out among the few timber species with commercially viable populations. In light of the current findings, it was recommended that:

Miombo woodlands should undergo a forest closure period corresponding to at least one cutting cycle for all merchantable timber species, especially the most desirable, except *B. spiciformis, B. boehmii*, and *J. globiflora*;

Commercial timber species should be reclassified to provide species of the genera *Brachystegia* and *Julbernardia* more value;

In addition to the minimum felling diameter, a minimum harvestable stem density for commercial-sized trees should be legally established for each timber species;

A timber species cannot be harvested if it exhibits any sort of rarity (geographical rarity, numerical rarity, or habitat specificity);

Forest logging for any timber species should be limited to miombo woodlands of the humid ecoregion.

## Data availability

The authors do not have permission to share data.

## Funding

The study did not receive any funding.

## Declaration of competing interest

The authors declare that they have no known competing financial interests or personal relationships that could have appeared to influence the work reported in this paper.

## Footnotes

1 Based on National Statistics Institute data and predictions of the rural population [13], excluding those in areas where miombo woodlands do not exist.
